# Environmental Persistence Influences Infection Dynamics for a Butterfly Pathogen

**DOI:** 10.1371/journal.pone.0169982

**Published:** 2017-01-18

**Authors:** Dara A. Satterfield, Sonia Altizer, Mary-Kate Williams, Richard J. Hall

**Affiliations:** 1 Odum School of Ecology, University of Georgia, Athens, Georgia, United States of America; 2 Biological Sciences, University of Arkansas at Little Rock, Little Rock, Arkansas, United States of America; 3 Department of Infectious Diseases, College of Veterinary Medicine, University of Georgia, Athens, Georgia, United States of America; Cary Institute of Ecosystem Studies, UNITED STATES

## Abstract

Many pathogens, including those infecting insects, are transmitted via dormant stages shed into the environment, where they must persist until encountering a susceptible host. Understanding how abiotic conditions influence environmental persistence and how these factors influence pathogen spread are crucial for predicting patterns of infection risk. Here, we explored the consequences of environmental transmission for infection dynamics of a debilitating protozoan parasite (*Ophryocystis elektroscirrha*) that infects monarch butterflies (*Danaus plexippus*). We first conducted an experiment to observe the persistence of protozoan spores exposed to natural conditions. Experimental results showed that, contrary to our expectations, pathogen doses maintained high infectivity even after 16 days in the environment, although pathogens did yield infections with lower parasite loads after environmental exposure. Because pathogen longevity exceeded the time span of our experiment, we developed a mechanistic model to better explore environmental persistence for this host-pathogen system. Model analysis showed that, in general, longer spore persistence led to higher infection prevalence and slightly smaller monarch population sizes. The model indicated that typical parasite doses shed onto milkweed plants must remain viable for a minimum of 3 weeks for prevalence to increase during the summer-breeding season, and for 11 weeks or longer to match levels of infection commonly reported from the wild, assuming moderate values for parasite shedding rate. Our findings showed that transmission stages of this butterfly pathogen are long-lived and indicated that this is a necessary condition for the protozoan to persist in local monarch populations. This study provides a modeling framework for future work examining the dynamics of an ecologically important pathogen in an iconic insect.

## Introduction

Many pathogens achieve transmission by being shed into the environment and persisting long enough to encounter and infect a new host. Scientists have long recognized environmental transmission, particularly among insect pathogens, with Louis Pasteur being among the early investigators to describe this strategy when a *Nosema* outbreak devastated the French silkworm industry in the 1860s [1]. Environmental transmission is now known to be common among diverse insect pathogens, including viruses, fungi, protozoa and nematodes [2]. Mathematical models have explored the influence of environmental transmission on invertebrate host and pathogen population dynamics and stability [3, 4, 5]. Despite these advances and the pervasiveness of this transmission mode, the ecological implications of environmental transmission are less well understood compared to direct transmission between hosts.

The window of opportunity for pathogen transmission depends crucially on the longevity of infectious stages in the environment. Thus, pathogen persistence outside the host—which can range from days to decades (e.g., [6, 7])–should strongly influence infection dynamics. In support of this, a model of nuclear polyhedrosis virus (NPV) infection in gypsy moths showed that viruses with reduced longevity caused less severe infections and shorter epidemics [8]. Numerous pathogens used for biological control of insects are environmentally transmitted, with some of the most effective having long persistence times [9]. Using genetic engineering or protective adjuvants to lengthen environmental persistence has improved the efficacy of some biological control agents, such as granulosis virus in codling moths [2]. Pathogen persistence can also affect transmission among beneficial insects; recent work on pathogens of pollinators showed that even strains with limited environmental longevity (surviving <3 hours) deposited onto flowers can lead to new protozoan and microsporidian infections in foraging bees [10]. As these studies highlight, understanding the extent and effect of pathogen persistence in the environment can help evaluate control strategies for pest insects and predict consequences of parasitism for beneficial insects.

Monarch butterflies (*Danaus plexippus*) and their protozoan pathogen *Ophryocystis elektroscirrha* (*OE*) provide a well described system that is useful for examining the consequences of environmental transmission. Infections develop internally in monarch larvae and pupae, and adult monarchs emerge covered with millions of dormant protozoan spores on their exterior [11]. Transmission occurs when infected butterflies shed infectious spores onto eggs or milkweed leaves and spores are consumed by monarch larvae [12]. For wild monarchs, this specialist pathogen can cause debilitating disease that can shorten adult lifespan, diminish flight performance, or cause death during eclosion [13, 14, 15]. Previous work suggests that *OE* disease removes some monarchs from the population during the butterflies’ long-distance migration from eastern North America [16]. This protozoan has been detected in all monarch populations examined to date, and prevalence varies by location, season, and year [17,18].

Monarchs in eastern North America migrate over 2500 km each fall to overwintering sites in central Mexico [19,20]. Migratory monarchs remain non-reproductive until the spring, when they return to the their breeding range to lay eggs on newly emerging milkweed plants [21]. During April-September, monarchs undergo 3 to 4 successive breeding generations that expand the monarchs’ range into the northern U.S. and southern Canada [22, 23]. The transmission of *OE*, which depends on monarch larvae ingesting spores, primarily occurs during the breeding season. Infection prevalence increases as the monarchs’ breeding season progresses and peaks just before the fall migration [16]. This pattern suggests that protozoan spores accumulate on milkweed host plants over time, and if these spores remain viable, monarchs born late in the season face a higher risk of infection than earlier cohorts.

*OE* spores have a thick, amber-colored wall that appears to offer some protection from environmental stress [11]. Previous work indicates that the protozoan spores remain infectious for several months under laboratory conditions [S. Altizer, unpublished data]. Spore longevity could be reduced, however, under natural conditions; spores shed onto milkweed could be damaged during exposure to UV light or high summer temperatures or could be displaced by wind or rain. Quantifying environmental persistence would allow researchers to better predict variation in *OE* infection risk across the monarch’s annual migratory cycle. This knowledge could be useful for evaluating the population impacts of a pervasive pathogen that often removes monarchs from the migratory population.

Here we designed a study to measure *OE* spore longevity under field conditions and investigate its effect on infection patterns. First, we experimentally tested spore decay in a natural setting. We deposited infectious parasite doses onto milkweed plants, exposed the plants to two outdoor environmental treatments (sun vs. shade), and measured pathogen infectivity and infection severity over time. Counter to our expectations, spore doses showed no significant loss of infectivity over the two-week span of the experiment, which limited our ability to quantify spore decay rate. However, environmental exposure of spore doses did produce infections with lower pathogen loads over time, thus providing evidence that some parasite spores were lost or killed in the environment. Second, we developed a mathematical model to better understand the role of parasite longevity in infection dynamics. While the experiment was not sufficiently long to provide parasite decay rate as a model parameter, we used our model to investigate how *OE* persistence time in the environment affects infection prevalence for monarchs during a typical summer-breeding season within a single milkweed patch. We also examined the effect of spore deposition rate on infection dynamics. Our modeling work indicated that environmental persistence of at least 3 weeks was necessary for pathogens to persist in the monarch population through the breeding season, and longer persistence times (5 to 12 weeks, or longer) were required for prevalence to reach levels commonly observed in the wild. Our study provides the first test of environmental persistence for this naturally occurring protozoan pathogen and introduces a mechanistic model to better understand key drivers of infection in this butterfly-pathogen interaction.

## Materials and Methods

### Environmental persistence experiment

#### Exposure of parasites to environmental conditions

We experimentally tested the longevity of *OE* spore doses following exposure to two environmental treatments (sun and shade) over 16 days. Prior lab findings demonstrated that *OE* spores are sensitive to ultraviolet radiation and heat [S. Altizer, unpublished data]. Wind and rain could also remove spores and further reduce host-parasite interactions. Thus, we predicted that both parasite infectivity of (the probability of infecting a susceptible host once contacted) and infection severity (quantitative pathogen load) would decrease with longer environmental exposure time, and that spores exposed to sunlight and rainfall would decay more rapidly than spores on shaded, sheltered plants.

To set up the experiment, we manually deposited pathogen spores onto marked leaves of greenhouse-grown, potted swamp milkweed plants (*Asclepias incarnata*). We obtained spores by swabbing lab-reared adult monarchs previously infected with one of three pathogen isolates. Isolates were originally derived from wild monarchs in eastern North America: Isolate E3 was collected in Cape May, NJ, isolate E10 from St. Paul, MN, and isolate E13 from Sweet Briar, VA. Parasite isolates were stored in individual envelopes in our laboratory at 12°C and were re-vived periodically through live monarchs to maintain viability; the most recent propagation was in March 2013. Isolates were chosen for this study to represent different levels of virulence (defined as parasite-induced reduction in host longevity as measured in [24]). Previous studies have documented variation among isolates for pathogen morphology and virulence [24, 25]. Additional details for isolates are provided in the S1 File.

To mimic the behavior of an infected female monarch shedding *OE* spores while ovipositing, we transferred approximately 200 spores, counted at 63 X magnification, onto the underside of each milkweed leaf using a glass wand (as described in [15]). We chose 200 spores as an initial dose for two reasons: First, this starting dose is within the range of the number of spores that a heavily infected female deposits onto a leaf during oviposition [26] and likely represents a realistic number of parasites that could be shed onto a wild milkweed leaf. Second, prior work showed that 100 ‘fresh’ spores caused a 100% infection rate in second-instar larvae [15]; we applied 200 spores because (i) our experiment used third-instar larvae, which are less susceptible than second-instars [S. Altizer, J. de Roode, M. Strand, unpublished data], (ii) we assumed that many of these spores would die or be lost from milkweed leaves prior to larval exposure, and (iii) we aimed to achieve high infectivity throughout the experiment to maintain sufficient sample sizes for measuring infection severity.

Our study was designed to compare parasites exposed to 0, 6, 11 or 16 days (time treatments) outdoors in sun or shade conditions (environmental treatments). We inoculated and marked 5 to 6 leaves per milkweed plant. Inoculated plants were either exposed to 0 days outdoors (N = 6 plants) or were placed outdoors in Athens, GA for 6, 11, or 16 days in a sun treatment on a grass lawn or in a shade treatment under a fiberglass screen enclosure with a tarp roof that shielded rainfall and direct sunlight (N = 36 plants, with 2 plants per each of 3 isolates per 6 outdoor treatment groups). The Day 0 leaves were fed to larvae the same day that spores were applied and were never placed outdoors. The Day 6, Day 11, and Day 16 leaves experienced outdoor temperatures during the experiment (June 6–21, 2014) ranging from 19 to 42°C in the sun treatment (average daily temperature 29.6°C) and 17 to 37°C in the shade treatment (average daily temperature 26.6°C). Six precipitation events occurred. Plants were kept in trays of water, and time and isolate treatment groups were spatially interspersed. Finally, we fed milkweed leaves without spores to 20 additional control monarchs to confirm that larvae were not acquiring infections due to laboratory contamination. These un-inoculated monarchs were not included in statistical analyses. No specific permissions were required for the land on which we conducted our outdoor experiment.

#### Assessment of parasite infectivity and infection severity

To test the infectivity of spores after environmental exposure, we fed individual inoculated leaves to lab-reared monarch larvae (1 leaf per larva) in the early third instar, when monarchs are large enough to consume an entire leaf yet still susceptible to *OE* infection. Larvae were kept in petri dishes for up to 48 hours until the leaf was consumed. Monarch larvae were grand-progeny of wild uninfected monarchs collected in Savannah, GA during spring 2014. Larvae that consumed the leaf were reared individually in 0.47L plastic containers with mesh screen lids under ambient light at average minimum and maximum temperatures of 27.4° and 30.8°C, respectively. Larvae were given fresh *A*. *incarnata* stalks daily. We reared a total of 200 larvae, with 25 larvae for each of six time-by-environment treatment groups, 30 larvae for the Day 0 plants, and 20 larvae for the control (uninoculated) group.

We recorded signs of *OE* infection during pupal development (following [24]). For monarchs that showed no sign of infection as pupae, we verified infection status after eclosion by pressing transparent tape (1.27cm^2^) against the butterfly’s abdomen and observing the sample for spores at 63X (following [16, 18]). *Infection status* (infected or uninfected) was noted for each monarch based on the presence of spores. We recorded sex and held all adults in individual glassine envelopes, prior to freezing at -20°C. Infectivity was calculated as the proportion of inoculated monarchs that became infected. We quantified parasite load of infected butterflies by vortexing each abdomen for 5 minutes in 5 mL deionized water and used a counting chamber to estimate the total number of spores per individual; this measure is hereafter referred to as *infection severity* [15]. Past work showed that more severe infections (with higher pathogen load) produce a greater reduction in adult monarch lifespan [15, 27].

We tested how environmental exposure affected monarch infection status and infection severity. First, we used a logistic regression to test the main effects of exposure time (0, 6, 11, or 16 days, treated as a continuous variable), exposure treatment (sun versus shade), and pathogen isolate on binary infection status (infected/uninfected). We used a general linear model to test the same main effects on infection severity (log-transformed quantitative pathogen load) among infected monarchs (with uninfected monarchs excluded). Because monarchs inoculated with spores in the Day 0 group were not placed in sun versus shade treatments, we randomly assigned sun or shade treatments to these individuals for the purposes of statistical analysis. This approach allowed us to assess all treatment groups in the same analyses across all experimental monarchs and time points. To ensure this approach did not alter experimental conclusions, we also ran the analyses without Day 0 monarchs and found similar results. Data from plants assigned to pathogen isolate E13 in the Day 6 exposure group (n = 17) were excluded from analyses, as these leaves did not receive the full pathogen dose due to experimental error. Analyses were conducted in R v. 3.0.3 [28].

### Model development

#### Construction and parameterization of model

**Fig 1:**
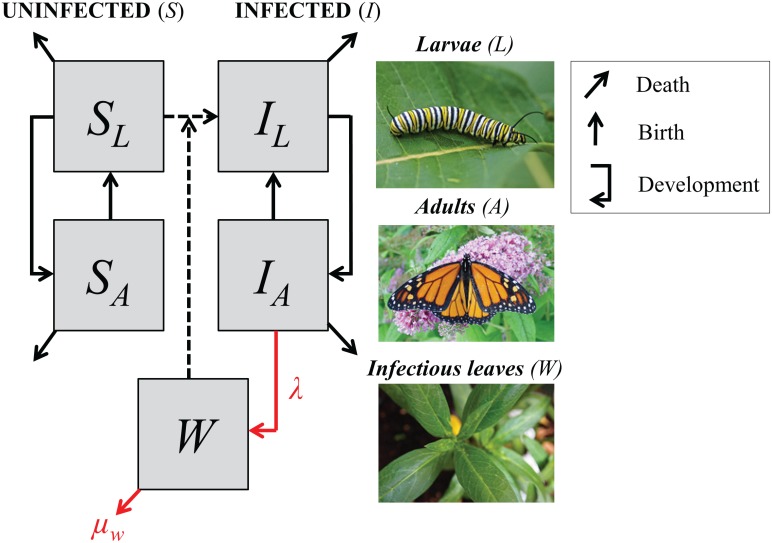
Schematic of model representing protozoan pathogen transmission in monarch hosts. Monarchs are represented as uninfected or infected immature stages (*S*_*L*_ or *I*_*L*_) or adults (*S*_*A*_ or *I*_*A*_). Milkweed leaves with spores (*W*) arise when infectious parasite doses are deposited by infected adult monarchs (at rate *λ*), and are lost through larval consumption (*c*) or through decay in dose viability (at rate *μ*_*W*_). The model assumes that all larvae produced by infected adult females (I_A_) are born with infections (I_L_; perfect vertical transmission). Uninfected larvae (S_L_) can become infected (I_L_) by consuming leaves with spores (environmental transmission, dashed line).

We next used a mechanistic model to examine the ecological consequences of environmental persistence of pathogens. We constructed a simple stage-structured model to describe host-parasite dynamics within a milkweed patch at a Midwestern site during a typical summer-breeding season (approximately 100 days, June-August). Monarch hosts were subdivided according to *OE* infection status and life stage, such that *S*_*L*_ and *I*_*L*_ represented the abundance of susceptible vs. infected pre-adult monarchs (larvae, eggs, and pupae) and *S*_*A*_ and *I*_*A*_ represented uninfected vs. infected adults (Fig 1). Pathogen infectious stages in the environment were described as the number of milkweed leaves with an infectious dose of spores (*W*). Experimental work has shown that doses of 10 to 100 spores are highly infectious causing between 70 to 100% of inoculated second instar larvae to acquire infection [15]. We assumed that monarch eggs were produced by adults at per capita rate *b* and developed into adults at rate *g*; we further assumed that monarch larvae experienced per capita density-independent mortality at rate *μ*_*0*_ and density-dependent mortality at rate *μ*_*1*_. Adults experienced per capita mortality at rate *μ*_*A*_.

Pathogen transmission was modeled via two pathways. *Vertical transmission* (from parent to offspring) occurs when infected adult monarchs transfer spores directly onto eggs or onto the milkweed surface surrounding the egg during oviposition; we modeled vertical transmission by assuming eggs laid by an infected female become infected. In this case, we assumed that vertical transmission occurs at rate *b* (the host birth rate) based on prior experimental work showing that infected female monarchs infect almost all, or over 90%, of their larvae [12]. *Environmental* or *horizontal transmission* (between unrelated hosts) occurs when susceptible larvae consume milkweed leaves contaminated with spores shed by unrelated adults. We modeled environmental transmission as the product of the larval consumption rate of milkweed leaves, *c*, and the probability of encountering contaminated leaves, *W*/*M* (where *M* is the total number of leaves in the milkweed patch). Fitness costs of parasitism occur after pupation and were modeled as a reduced probability of eclosion and reproduction, *p*_*E*_, and increased adult mortality rate, *μ*_*I*_, relative to uninfected adults. Under these assumptions, host-parasite dynamics in hosts were described by the following system of ordinary differential equations:

$$\frac{\text{d}S_{L}}{dt}=\;bS_{A}-\left(\mu_{0}+\mu_{1}\frac{S_{L}+I_{L}}{M}\right)S_{L}-gS_{L}-\frac{cW}{M}S_{L}$$

$$\frac{\text{d}I_{L}}{dt}=\;bI_{A}-\left(\mu_{0}+\mu_{1}\frac{S_{L}+I_{L}}{M}\right)I_{L}-gI_{L}+\frac{cW}{M}S_{L}$$

$$\frac{\text{d}S_{A}}{dt}=\;gS_{L}-\mu_{A}S_{A}$$

$$\frac{\text{d}I_{A}}{dt}=\;p_{E}gI_{L}-\mu_{I}I_{A}$$

We assumed that leaves are exposed to infectious doses when adults deposit spores while nectaring or ovipositing on milkweed plants at rate *λ*. Leaf exposure also depended on the probability that a visited leaf did not already have spores (1-*W*/*M*). Two processes removed pathogens from milkweed. Spores could die or fall off, causing environmentally exposed leaves with parasite doses to be eliminated at rate *μ*_*w*_. Additionally, exposed leaves were consumed by larvae at a rate proportional to larval abundance (*S*_*L*_+*I*_*L*_), the larval consumption rate *c*, and the probability that the leaf consumed had spores (*W*/*M*). Therefore, the dynamics of the environmental stages of *OE* were described as

$$\frac{\text{d}W}{dt}=\lambda \left(1-\frac{W}{M}\right)I_{A}-\mu_{w}W-\frac{cW}{M}\left(S_{L}+I_{L}\right)$$

Model parameters are outlined in Table 1 and details on parameter derivation appear in the S2 File.

**Table 1:** Parameters of the model, including definitions, units, and values. See the S2 File for derivation and data sources for parameter estimates.

Parameter	Definition	Units	Value
*b*	Host fecundity rate	eggs/adult/day	15
*g*	Immature host development rate (egg to adult)	1/day	0.0385
*c*	Larval consumption rate of milkweed	leaves/day	1.35
*p*_*E*_	Probability of infected larva eclosing and mating successfully		0.72
*μ*_*0*_	Density-independent *per capita* larval mortality rate	1/day	0.08
*μ*_*1*_	Density-dependent *per capita* larval mortality rate based on a larval density of 0.25 larvae/plant	1/day	1372
*μ*_*A*_	Mortality of uninfected adult monarchs	1/day	0.0417
*μ*_*I*_	Mortality rate of infected adult monarchs	1/day	0.05
*λ*	Shedding rate of infectious doses onto leaves (per adult)	leaves/day	1–300
*μ*_*w*_	Decay rate of infectious doses on milkweed leaves	1/day	0.0125–1.0
*S*_*0*_	Initial uninfected adult monarch population size	adults	18
*I*_*0*_	Initial infected monarch population size	adults	2
*M*	Total number of milkweed leaves in patch	leaves	25000
*T*	Length of breeding season	days	100

#### Exploration of model sensitivity to rates of parasite decay and deposition

Whereas estimates of most parameters in our model were derived from previous studies or personal observations, the pathogen dose decay rate *μ*_*w*_ in the environment and the pathogen shedding rate (*λ*) remain unknown. Our experiment, while originally designed to estimate pathogen persistence, was not long enough to detect decay rate of pathogen doses. Thus, we conducted a sensitivity analysis, varying the duration of environmental pathogen persistence (1/*μ*_*w*_, or the inverse of pathogen decay rate) from 1 to 80 days and the pathogen shedding rate (*λ*) from 1 to 300 leaves per day per adult monarch. We expected that infected monarchs might shed spores on up to 300 leaves per day, based on observations of wild monarchs visiting an average of 70 milkweed stalks per hour while nectaring or ovipositing in a garden in Georgia [A. Majewska, personal communication]. We observed effects on infection prevalence and host population size during the 100-day breeding season. We used the *deSolve* package in R v. 3.0.3 to solve the system of differential equations [28].

## Results

### Spore persistence on milkweed leaves

The vast majority (90%) of inoculated and un-inoculated monarchs survived to adulthood. Among inoculated monarchs, 74% acquired *OE* infections. Average pathogen load for infected monarchs was 10^5.55^ spores (± 10^4.43^ SEM). No un-inoculated monarchs became infected.

**Fig 2:**
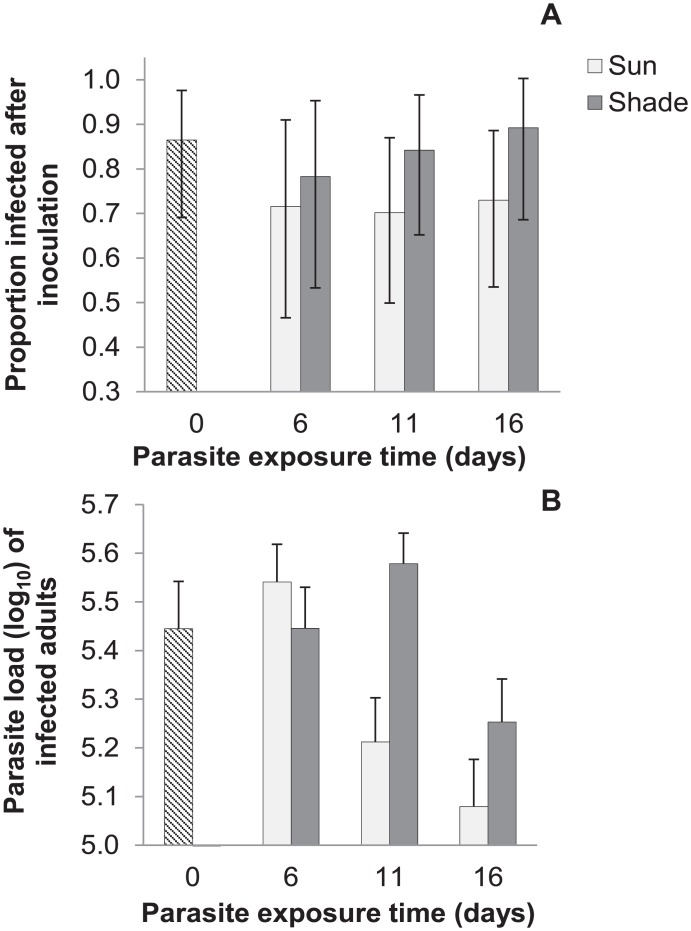
Experimental results on infectivity and pathogen load following spore exposure to sun or shade treatments for 0, 6, 11 or 16 days. (a) Proportion of monarchs infected following inoculation with spores on milkweed leaves exposed for 0 days (n = 27); 6 days, in either sun (n = 15) or shade conditions (n = 15); 11 days in sun (n = 23) or shade (n = 24); or 16 days in sun (n = 25) or shade (n = 19). Error bars show Jeffrey’s 95% confidence intervals for binary data. (b) Pathogen load (log-transformed) of monarchs infected with spores exposed for 0 days (n = 22); 6 days, in either sun (n = 10) or shade conditions (n = 11); 11 days in sun (n = 14) or shade (n = 18); or 16 days in sun (n = 17) or shade (n = 16). Error bars show standard error of the mean.

Exposure time did not significantly affect infectivity (probability of infection; χ^2^ = 0.16, df = 1, p = 0.69, NS). Pathogen doses exposed for 16 days in the environment infected a similar proportion of inoculated monarchs (0.75) as pathogen doses exposed for 0 days (0.81 of inoculated monarchs; Fig 2A). Thus, our experiment showed that spores remained highly infectious even after 16 days in the natural environment. There was also no significant difference in infectivity between sun and shade exposure treatments, although infectivity did tend to be lower for spores exposed to the sun compared to the shade (Fig 2A; χ^2^ = 1.6, df = 1, p = 0.21, NS). Infection probability varied for the three pathogen isolates, with isolate E3 resulting in significantly higher infection rates (0.90 across all larvae) compared to isolate E10 (0.64 infection rate; χ^2^ = 9.7, df = 2, p = 0.008).

Among the subset of infected monarchs, infection severity (measured as total pathogen load) declined with greater exposure time to the environment (Fig 2B; F_1,106_ = 6.96, p = 0.01). This suggests that some spores on each inoculated leaf lost viability during environmental exposure, resulting in smaller effective doses (details appear in the S1 File). Spores exposed to the sun caused infections with lower parasite loads compared to spores from the shade treatment (Fig 2B; F_1,82_ = 4.33, p = 0.04). Pathogen load varied among isolates but this effect was non-significant (F_2,82_ = 2.74, p = 0.07, NS).

### Infection dynamics and effects of parasite persistence and deposition

**Fig 3:**
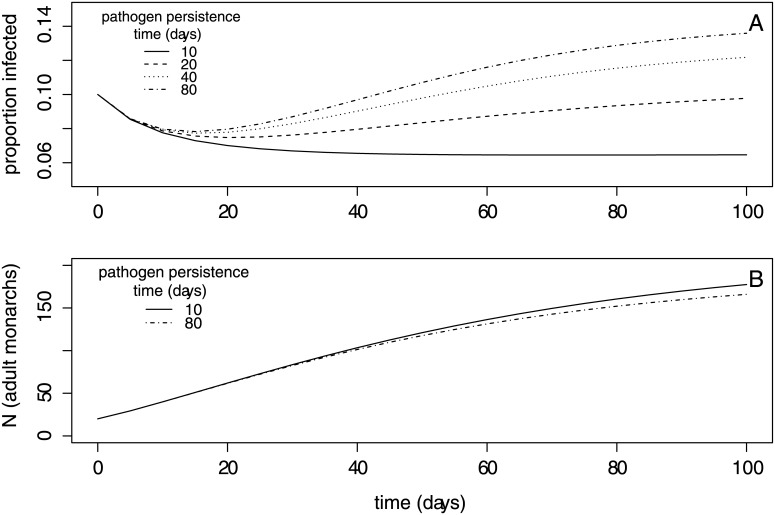
Results from the transmission model showing within-season dynamics. Within-season dynamics in (a) infection prevalence and (b) adult population size within the milkweed patch for a range of pathogen persistence times (in days, 1/*μ*_*w*_). Longer pathogen persistence produced higher infection prevalence and slightly lower adult population size. The dynamics shown assume an intermediate value for parasite shedding rate (*λ* = 150 leaves/day/adult); all other parameter values are listed in Table 1.

We used the model to first examine how host population size and infection prevalence varied over time within a breeding season in relation to pathogen persistence (1/*μ*_*W*_) and shedding rate (*λ*). For moderate to high environmental persistence times (1/*μ*_*W*_ > 20 days) and pathogen shedding rate (*λ* > 150 leaves/day/monarch), our model captured the steady increase in prevalence observed among wild summer monarchs as the breeding season progresses [16]. Similar to wild data, our model showed that prevalence peaked at approximately 15% at the end of the season (Fig 3A). Abundance of monarch larvae and adults increased throughout the breeding season, and declined weakly with greater pathogen environmental persistence (Fig 3B).

**Fig 4:**
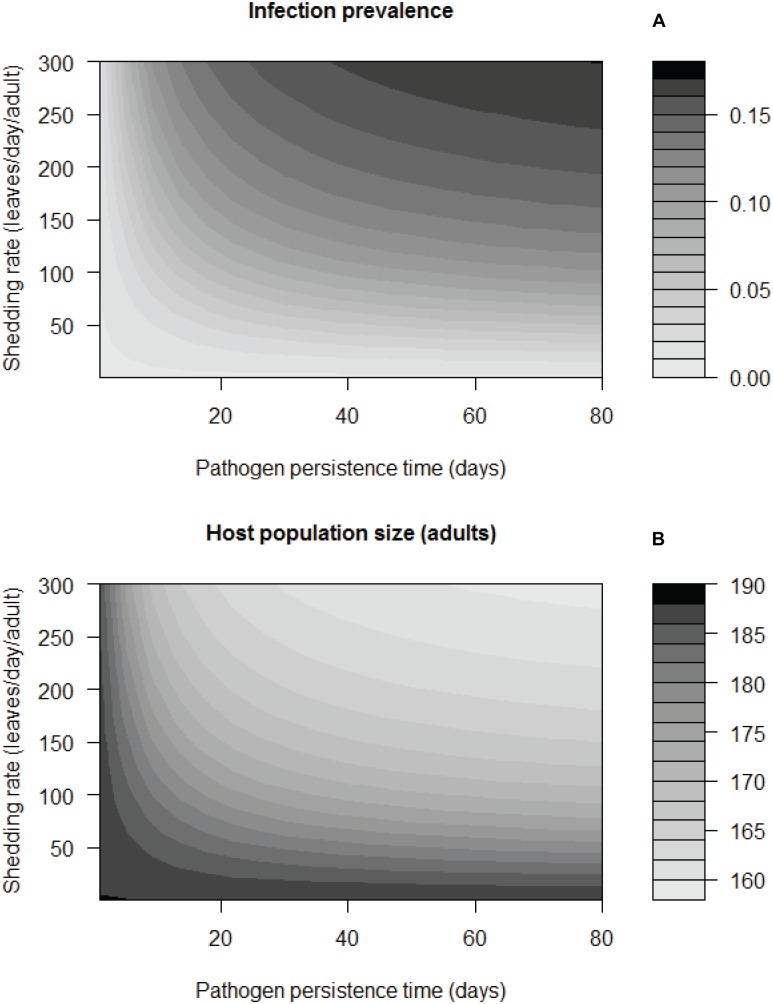
Parasite prevalence and host population size at the end of the season based on the transmission model, for a range of pathogen persistence times (1/*μ*_*W*_) and shedding rates (*λ*). Results from the model shown as heat contours for (a) infection prevalence at the end of the breeding season, and (b) adult monarch population size (T = 100 days; scale bars), depending on a range of pathogen persistence times and shedding rates.

We quantified how final infection prevalence at the end of the breeding season depended on the duration of environmental persistence (1/*μ*_*W*_; Fig 4). When assuming a moderate pathogen shedding rate (*λ* = 150 leaves/day), model analyses showed that *OE* spores must persist on milkweed leaves for a minimum of 22 days for infection prevalence to increase above initial conditions (Fig 4A), and spores must persist for more than 80 days for prevalence to reach values observed in wild monarchs by the end of the breeding season [16]. When assuming a higher value for pathogen shedding rate (*λ* = 250 leaves/day), pathogen spores must persist for 40 days for prevalence to reach the upper range of values observed in the wild (Fig 4A). In general, prevalence at the end of the season increased as environmental persistence of pathogens increased. However, beyond a persistence time of approximately 50 days (depending on other parameter values), the end-of-season prevalence remained similar (Fig 4A), likely because the proportion of contaminated milkweed leaves in the patch (*W/M*) saturates at around 80%. Longer spore persistence in the environment mildly decreased final adult population size, such that the adult population was 10–15% smaller when pathogens were long-lived (e.g., persist 50 days) compared to when pathogens were short-lived (Fig 4B).

Other factors beyond spore persistence were important for host-pathogen dynamics. Although the shedding rate in the wild of infectious doses onto milkweeds (*λ*) is not known, this parameter can influence infection dynamics (Fig 4). High shedding rates increased infection prevalence at the end of the season (Fig 4A) and the rate at which prevalence rose during the breeding season (Fig 3A). High shedding rates (e.g., *λ* = 300 leaves/day) also reduced the influence of spore persistence on end-of-season infection prevalence, such that even infectious doses persisting for approximately 24 days resulted in late-season prevalence greater than 15% (Fig 4A).

## Discussion

Our study suggested that transmission stages of *Ophryocystis elektroscirrha* remain infectious for multiple weeks in natural conditions, and that spore persistence in the environment is essential for *OE* to invade and persist in local monarch populations. Experimental results showed that pathogen doses remained highly infectious after 16 days of environmental exposure. While the experiment did not allow us to measure pathogen decay quantitatively, a reduction in infection severity (i.e., pathogen load) over time suggested that environmental exposure killed or removed some pathogens within each dose. We developed a transmission model to examine how infection prevalence dynamics within a single breeding season responded to a range of pathogen persistence values. The transmission model indicated a threshold for environmental persistence above which the pathogen can invade and prevalence will increase during the summer-breeding season. Spore doses must remain viable for a minimum of 24 days (assuming maximum parasite shedding rate) to >80 days (assuming a moderate parasite shedding rate) to allow prevalence to reach the upper range of that observed in summer-breeding milkweed patches in eastern North America [16]. In addition to pathogen persistence, pathogen shedding rate by infected adult monarchs was an important determinant of late-season prevalence.

Previous work on the dynamical consequences of environmental persistence for insect pathogens showed that persistent environmental transmission stages can cause population cycles on a multiyear scale (e.g., [29, 30, 4]). Our study provides evidence that environmental persistence (modeled as 1/*μ*_*w*_) also influences short-term patterns in infection within a single season. Specifically, the model showed that parasites with longer environmental persistence led to higher prevalence and reached these levels earlier in the breeding season, compared to parasites with shorter environmental persistence time. The model also showed, for moderate levels of parasite persistence time, a general increase in infection prevalence as the breeding season progressed, comparable to patterns in the wild [16]. This within-season increase in infection prevalence was driven by an increase in the proportion of leaves contaminated with spores (model as *W/M*), indicating that spore longevity enables pathogens to accumulate on milkweed leaves, as previously suggested by field and experimental work [16].

Like many entomopathogens, *OE* can spread through both vertical and environmental (horizontal) transmission. Our model suggests contrasting roles for these modes of transmission that sustain monarch-*OE* interactions. Here, we found that environmental transmission (modeled in the c*W/M term) was critical to pathogen persistence within a single season. When environmental transmission was 0, vertical transmission alone (modeled in the *bI*_*A*_ term, representing parent-to-offspring transmission) was not sufficient for the pathogen to establish in the host population within season, consistent with prior modeling work [31, 32]. However, the biology of this system suggests that vertical transmission is likely crucial for long-term *OE* persistence *between* breeding seasons, when monarchs overwinter in Mexico before resuming reproduction in the spring. Native milkweed plants typically die back in the fall each year, a process that incidentally cleanses monarch habitat of *OE* spores (which are then lost in the soil). Thus, only those spores that survive on the exterior of the overwintering adult monarchs’ bodies for several months can be successfully transmitted (vertically) to the next generation in the spring. We expect that the monarchs’ migratory cycle thus selects for even greater spore longevity, compared to populations of monarchs that breed year-round. Consequently, both vertical and environmental transmission require pathogen spores to persist for substantial periods of time, with vertical transmission causing new infections across years and environmental transmission contributing to pathogen increase during the breeding season.

Our model emphasized that the rate at which infected adult monarchs shed pathogens onto leaves (*λ*), as determined by butterfly visitation of milkweed leaves, strongly increased infection prevalence. This could have ecological consequences at the landscape level, which could be tested in future work. We expect parasite shedding rate to be higher when monarchs visit milkweed plants more frequently, such as in areas where monarchs rely heavily on milkweed plants not only for ovipositing but also as the primary nectar source. Such interactions could occur particularly in coastal areas of the southern U.S., where some monarchs continue breeding and nectaring on exotic milkweeds into the winter [33] when other nectar sources are limited. Conversely, locations with a high diversity of nectar sources could lower milkweed visitation rates and decrease opportunities for pathogen spread.

Although the experimental results motivated our model development, we did not observe a decline in infectivity of parasite doses on leaves as predicted over two weeks of environmental exposure, precluding the opportunity to parameterize pathogen decay rate in the model. However, some degree of pathogen decay was supported, as spore doses exposed to the environment caused infections with lower final pathogen loads. This suggests that the number of viable spores declined through time due to increased exposure to harmful abiotic conditions. The presumed loss of viable spores was more apparent among sun-exposed pathogen doses than shade-exposed pathogens, consistent with previous laboratory work showing that UV exposure can destroy protozoan spores [S. Altizer, unpublished], a phenomenon that is well described for many other insect pathogens [9, 34].

One assumption of our model is that all infected monarchs transmit pathogens at the same rate and experience the same costs of infection. However, prior field and laboratory work showed that pathogen load per butterfly varies among infected monarchs, and that pathogen load influences pathogen shedding, the probability of infection for larvae consuming the spores, and infection severity [15]. Thus, heterogeneity in spore load on infected adults and spore dose consumed by susceptible caterpillars could affect infection outcomes. For example, monarchs with higher spore loads may shed larger infectious doses onto milkweed and thus contribute disproportionately to transmission (i.e. act as superspreaders *sensu* [35]), potentially accounting for the high prevalence observed in some wild monarch populations in North America [18] beyond that predicted by our model. To fully explore the consequences of such heterogeneities would require a more sophisticated, individual-based modeling approach; first, however, further empirical work is needed to quantify key unknowns, such as the relationship between spore load in adult monarchs and the number of spores shed onto milkweed leaves. These biologically-informed initial spore doses could then be used in longer-running experiments to estimate pathogen decay rate (*μ*_*W*_) under natural conditions. Better estimates of milkweed visitation rates of wild monarchs are also needed, as pathogen shedding rate (λ) was shown to strongly influence end-of-season infection prevalence.

Migratory monarchs in eastern North America have recently experienced severe declines, mostly attributed to habitat loss at breeding and overwintering sites [36, 37,38]. The extent to which mortality associated with pathogen infection has affected monarch population declines remains unclear. Our model predicted that mild reductions in the number of late-breeding season adults (on the order of a 16% reduction) could result from higher pathogen shedding and spore persistence, an effect that was likely limited by the relatively low infection prevalence observed in the model. Conditions that might crowd larvae, such as habitat fragmentation, could further increase prevalence and lead to stronger pathogen-mediated declines. Importantly, recent field monitoring documented extremely high infection prevalence in year-round breeding patches in the southern U.S. [33], a result not captured by our within-season model that extended to only 100 days. Further work integrating empirical data and modeling approaches is needed to understand drivers of spatial heterogeneity in infection and to predict future pathogen impacts on wild monarch populations.

Beyond monarchs, our study contributes to a growing body of work that highlights how pathogen persistence in the environment influences infectious disease dynamics across diverse systems. Modeling approaches demonstrated that environmental transmission enables the spread and persistence of avian influenza in water bird populations [39], causes population cycling of red grouse controlled by nematode infections [40], and enhances the persistence of hantavirus in wild rodents [41]. There is increased interest in modeling environmental transmission of pathogens [42] in the face of global environmental change. This is particularly relevant to insect pathogens, including vector-borne pathogens, where host and pathogen survival and distribution depends critically on temperature and precipitation [43, 44].

## Supporting Information

S1 File Experimental details: Pathogen isolates and spore load. (DOCX)

S2 File Model parameters. (DOCX)

S3 File Experimental data. (CSV)
