# Supplementary material for: Environmental Persistence Influences Infection Dynamics for a Butterfly Pathogen
Source: PLoS One. 2017 Jan 18;12(1):e0169982. doi: 10.1371/journal.pone.0169982 (PMC5242512; doi:10.1371/journal.pone.0169982)
Supplement: S1 File — (DOCX) [file pone.0169982.s001.docx]

**S1 Supporting Information**

*To accompany: Environmental Persistence Influences Infection Dynamics for a Butterfly Pathogen (Satterfield, Altizer, Williams, and Hall 2016)*

**Origin of pathogen isolates**

We chose three pathogen isolates for our environmental persistence experiment. Isolates E3, E13, and E10 were selected to represent low, moderate, and high levels of virulence, respectively, as documented in a previous experiment [1]. Virulence was measured as the inverse of the lifespan of infected adult monarchs; shorter lifespan indicated higher virulence. Isolates were originally collected from wild monarchs in eastern North America, with isolate E3 collected from Cape May, NJ in Oct. 2001; isolate E13 from Sweet Briar, VA in July 2005; and isolate E10 from St. Paul, MN in July 2005. Isolates have been passed through live monarchs to propagate spores multiple times since collection, with the most recent parasite propagation in March 2013. Isolates were stored at 12°C until we exposed spores to outdoor conditions in our experiment. Prior work suggested that spores can remain viable for long periods at 12°C. The high rate of infection in our experiment even within the control treatment (not exposed to outdoor conditions) indicated the spores used in our study were viable [S. Altizer, unpublished].

**Experimental Results: Pathogen load**

We measured pathogen load of infected butterflies at the end of our experiment as a proxy of *infection severity.* Lower spore loads indicate less severe infections. Among infected monarchs, total pathogen load declined with greater exposure time to the environment.


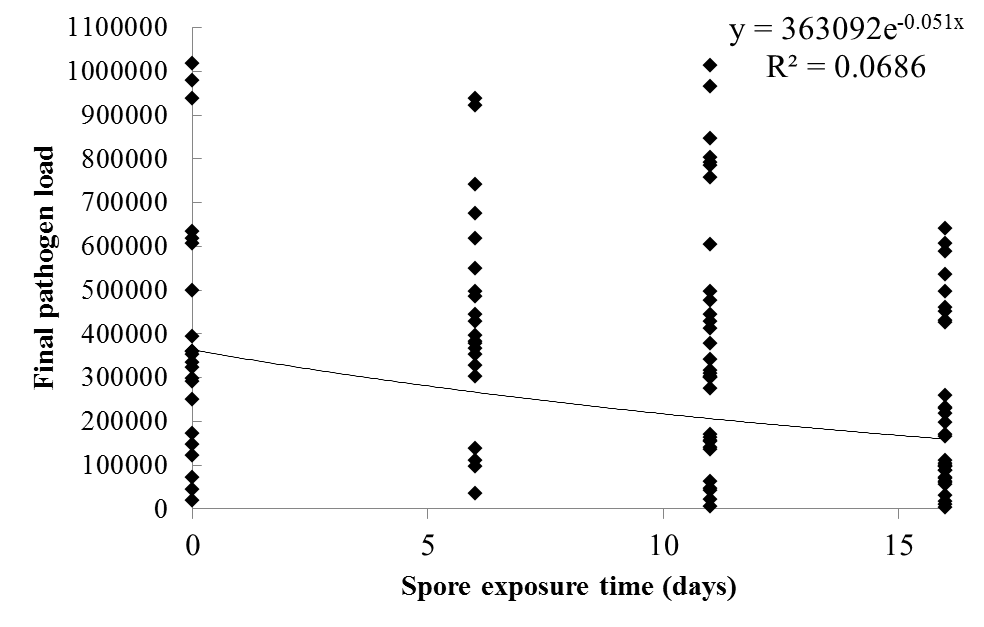


**S1 Fig 1. Exponential decay curve fit to pathogen load data over time.**

**References**

1. de Roode JC, Yates AJ, Altizer S. Virulence-transmission trade-offs and population divergence in virulence in a naturally occurring butterfly parasite. Proc Natl Acad Sci U S A. 2008 May 27;105(21):7489–94.
