# Supplementary material for: Environmental Persistence Influences Infection Dynamics for a Butterfly Pathogen
Source: PLoS One. 2017 Jan 18;12(1):e0169982. doi: 10.1371/journal.pone.0169982 (PMC5242512; doi:10.1371/journal.pone.0169982)
Supplement: S2 File — (DOCX) [file pone.0169982.s002.docx]

**S2 Supporting Information**

*To accompany: Environmental Persistence Influences Infection Dynamics for a Butterfly Pathogen (Satterfield, Altizer, Williams, and Hall 2016)*

**Model parameterization**

We represent the number of monarchs in pre-adult stages (egg, larvae, and pupae) in model compartments *S_L_* (susceptible) and *I_L_* (infected). Monarchs in the adult stage are represented in the *S_A_* (uninfected) and *I_A_* (infected) compartments. Because monarchs are among the most well studied invertebrates in the natural world, we can derive most parameter values for our model from the published literature. We use the degree-day model (described in [1]) to calculate development time for wild monarchs experiencing typical temperatures in the summer-breeding range: At 25.6°C (an average low daily temperature for June in Pennsylvania), breeding monarchs develop from eggs to eclosion in 26 days. Thus, we assume larval development in our transmission model occurs at a rate of *g*=1/26.

Previous work using outdoor cages showed that uninfected adult female monarchs live 24 days on average and infected adult females live 20 days on average [2]. Based on this study, our model assumes adult monarch mortality occurs at *μ_A_*=1/24 for uninfected individuals and *μ_I_*=1/20 for infected individuals.

We assume larvae (*S_L_* and *I_L_*) are produced at per-capita fecundity rate *b*. Past work showed that females produce an average of 715 eggs over their lifespan [3]. Assuming a 24-day adult lifespan and multiplying by ½ to only consider females in the population, we represent fecundity as *b*=15 eggs per day.

We represent larval consumption rate *c* as the total number of *Asclepias incarnata* milkweed leaves consumed per monarch. As larvae eat approximately 35 leaves over the 26-day time span from egg to eclosion [Satterfield, personal observations], we use *c*=1.35 leaves/day in the model.

We estimate the probability for infected monarchs successfully eclosing and reproducing, *p_E_,* using experimental data from infected monarchs in captivity [4]: Monarchs with an average pathogen load of 10^5.75^ spores had a 0.9 probability of eclosing and a 0.8 probability of mating, yielding *p_E_*=0.72.

Per capita larval mortality includes both density-dependent and density-independent components. In the absence of density dependence, the density-independent mortality rate *µ_0_* is related to the probability of surviving the 26-day period from egg to eclosion, *s*, such that

$$s=e^{-\mu0*26}$$

Rearranging the equation to solve for density-independent mortality gives:

$$\mu_{0}=- \frac{\ln s}{26}$$

Survival from egg to pupation has previously been reported as 0.12 [5] and is consistent with other estimates of survival [6]. Assuming *s*=0.12 yields *µ_0_*=0.08 for density-independent larval mortality rate. To estimate density-dependent mortality *µ_1_*, we first describe host dynamics in the absence of disease using a system of differential equations for number of larvae (*N_L_*) and number of adults (*N_A_*):

$$\frac{dN_{L}}{dt}= bN_{A}-\left. {\left( \mu_{0}+\mu_{1}\frac{N_{L}}{M} \right)N}_{L}-g \right.N_{L}$$

$$\frac{dN_{A}}{dt}= gN_{L}-{\mu_{A}N}_{A}$$

We can then solve the system of equations for *µ_1_* at equilibrium, when *dN_L_/dt* and *dN_A_/dt* are equal to 0:

$$\mu_{1}=\left( \frac{M}{N_{L}} \right) \left. \left( \frac{bg}{\mu_{A}} {- \mu}_{0}-g \right) \right.$$

We assume equilibrium larval density is 0.25 larvae/plant, based on the upper range of densities documented during the summer-breeding season in wild milkweed patches in the Midwest [6]. This places larval density per milkweed leaf at 0.01, assuming *A. incarnata* plants have an average of 25 leaves each [Satterfield, personal observation], making *M/N_L_*=100 in the expression above. We use this and the parameter values previously described to obtain density-dependent larval mortality rate *µ_1_*_=_1372*.*

We incorporate pathogen environmental stages into the model by representing the number of pathogen-exposed milkweed leaves as *W*. Transmission of OE pathogens occurs when infected adult monarch deposit spores onto milkweed leaves and larvae consume the spores before they become inviable. However, little is known about the deposition and decay of OE pathogens on milkweed leaves in natural settings. Thus, we vary spore shedding rate *λ* (1<*λ*<300 leaves/day/infected adult) and pathogen environmental persistence (1<$\frac{1}{\mu_{W}}$<80 days) in the model and observe effects on infection prevalence and adult abundance. Our experimental findings suggested spores commonly persist at least 16 days and thus have the potential to persist for longer periods. Spore shedding rate *λ* could reasonably occur at a rate between 1 and 300 leaves/day/infected monarch, based on observations that breeding monarchs can visit up to 70 milkweed stalks per hour and remain active for several hours per day [A. Majewska, personal communication].

We set initial conditions in the model to represent a milkweed patch early in the breeding season – when no larvae are present (*S_L_*=0 and *I_L_*=0), when newly sprouted milkweed leaves are abundant in the patch (*M*=25000 leaves assuming a patch of 1000 plants with 25 leaves/plant), and when no leaves have been exposed to pathogens (*W_0_*=0). We initially allow 18 uninfected adult monarchs (*S_A_*=18 at T=0) and 2 infected adult monarchs (*I_A_*=2 at T=0) to colonize the milkweed patch. This assumes an initial infection prevalence of 10%, as observed in samples from wild monarchs collected by citizen scientists in the *Monarch Health* program ([www.monarchpathogens.org](http://www.monarchparasites.org)). Specifically, *Monarch Health* samples from 1142 wild monarchs in the summer-breeding range collected early in the breeding season (April-July) in 2011-2014 indicate that early-season prevalence ranges from 5% to 20%, with an average of 12% across years. To use whole numbers of monarchs, we assumed 10% of monarchs were initially infected in our model.

**References**

1. Zalucki MP. Temperature and rate of development in Danaus plexippus L. and D. chrysippus L. (Lepidoptera: Nymphalidae). Aust J Entomol. 1982 Nov 1;21(4):241–6.

2. Altizer SM, Oberhauser KS. Effects of the Protozoan Parasite Ophryocystis elektroscirrha on the Fitness of Monarch Butterflies (Danaus plexippus). J Invertebr Pathol. 1999 Jul;74(1):76–88.

3. Oberhauser KS. Fecundity, lifespan and egg mass in butterflies: effects of male-derived nutrients and female size. Funct Ecol. 1997 Apr 1;11(2):166–75.

4. de Roode JC, Yates AJ, Altizer S. Virulence-transmission trade-offs and population divergence in virulence in a naturally occurring butterfly parasite. Proc Natl Acad Sci U S A. 2008 May 27;105(21):7489–94.

5. Borkin SS. Notes on shifting distribution patterns and survival of immature Danaus plexippus (Lepidoptera: Danaidae) on food plant Asclepias syriaca. Gt Lakes Entomol. 1982;15:199–207.

6. Prysby MD, Oberhauser KS. Temporal and geographic variation in monarch densities: Citizen scientists document monarch population patterns. In: The Monarch Butterfly: Biology and Conservation. Ithaca, NY: Cornell University Press; 2004.
